# The global geography of human subsistence

**DOI:** 10.1098/rsos.171897

**Published:** 2018-09-26

**Authors:** Michael C. Gavin, Patrick H. Kavanagh, Hannah J. Haynie, Claire Bowern, Carol R. Ember, Russell D. Gray, Fiona M. Jordan, Kathryn R. Kirby, Geoff Kushnick, Bobbi S. Low, Bruno Vilela, Carlos A. Botero

**Affiliations:** 1 Human Dimensions of Natural Resources, Colorado State University, Fort Collins, CO, USA; 2 Department of Linguistic and Cultural Evolution, Max Planck Institute for the Science of Human History, Jena, Germany; 3 Department of Linguistics, Yale University, New Haven, CT, USA; 4 Human Relations Area Files, Yale University, New Haven, CT, USA; 5 Department of Anthropology and Archaeology, University of Bristol, Bristol, UK; 6 Department of Ecology and Evolutionary Biology, University of Toronto, Toronto, Canada; 7 School of Archaeology and Anthropology, Australian National University, Canberra, Australian Capital Territory, Australia; 8 School for Environment and Sustainability, University of Michigan, Ann Arbor, MI, USA; 9 Department of Biology, Washington University, St Louis, MO, USA

**Keywords:** agriculture, animal husbandry, biogeography, foraging, subsistence

## Abstract

How humans obtain food has dramatically reshaped ecosystems and altered both the trajectory of human history and the characteristics of human societies. Our species' subsistence varies widely, from predominantly foraging strategies, to plant-based agriculture and animal husbandry. The extent to which environmental, social and historical factors have driven such variation is currently unclear. Prior attempts to resolve long-standing debates on this topic have been hampered by an over-reliance on narrative arguments, small and geographically narrow samples, and by contradictory findings. Here we overcome these methodological limitations by applying multi-model inference tools developed in biogeography to a global dataset (818 societies). Although some have argued that unique conditions and events determine each society's particular subsistence strategy, we find strong support for a general global pattern in which a limited set of environmental, social and historical factors predicts an essential characteristic of all human groups: how we obtain our food.

## 1. Introduction

Biogeography has advanced our understanding of how environmental conditions, geographical constraints and evolutionary history have shaped the abundance and phenotypes of species, as well as the diversity of biological communities [1]. However, few of the theoretical and methodological developments of this field have been applied to the study of humans [2–4]. One promising area of overlap is the study of diet. The possible connections between diet, species' ranges and geographical patterns of diversity have been examined in a wide range of non-human taxa [5–7]. In addition, biogeographers have debated the degree to which environmental productivity and stability, behavioural characteristics (e.g. hunting techniques), and phylogeny may determine diet [5–10]. Although similar environmental, social and historical constraints have been proposed to apply to humans, the degree to which these factors shape human subsistence strategies remains contested.

**Figure 1.**
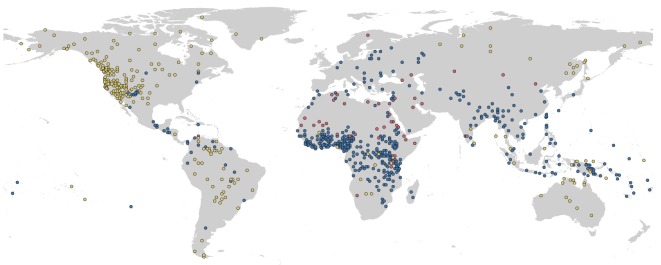
Global variation in dominant subsistence strategy for 818 societies. Yellow points, foraging; blue, plant-based agriculture; red, animal husbandry. See Material and methods for details on the sample.

Subsistence strategies are associated with many fundamental characteristics of human societies, including demography, settlement patterns, social and political organization and religious beliefs [11–14]. For most of human history, our species exclusively foraged for food via hunting, gathering and/or fishing [15]. The subsequent spread of food production as a primary mode of subsistence has been referred to as ‘the most important process in Holocene human history’ [11]. However, subsistence focused on plant-based agriculture has not been universally adopted. Even recently, hundreds of societies have maintained foraging as their primary mode of subsistence [16]. Although most extant human groups use multiple subsistence strategies, the dominant strategy within a group varies widely across the planet [17] (figure 1, see Material and methods for calculations).

**Figure 2.**
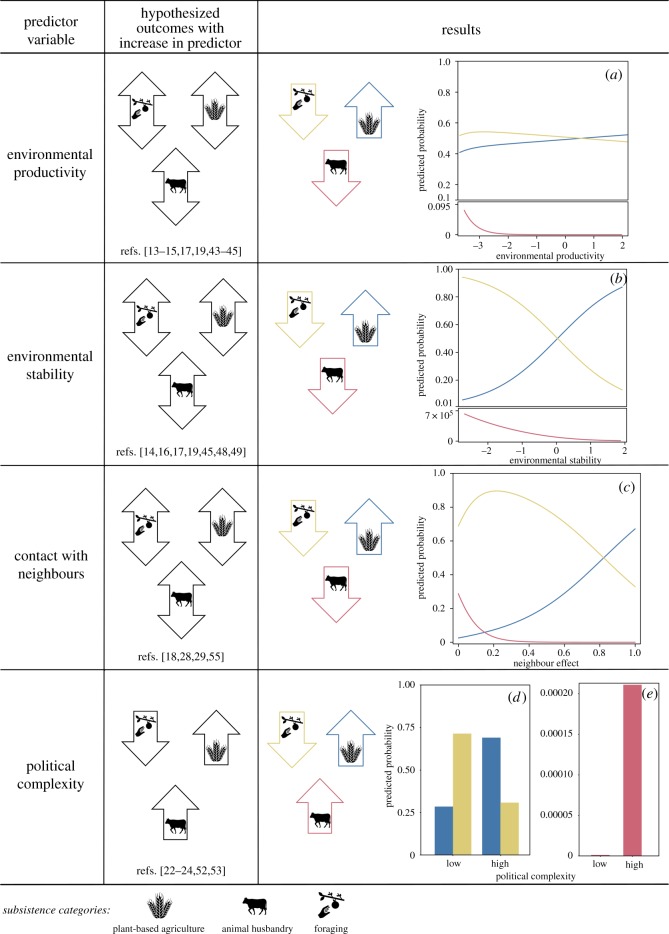
Hypothesized and observed effects of predictor variables on subsistence strategies. Down arrows indicate a decreased likelihood of subsistence strategy, up arrows indicate increased likelihood, bi-directional arrows indicate hypotheses proposed for both an increased and decreased likelihood. Predicted probabilities of dominant subsistence strategies (based on multi-model average results) varied with differences in environmental productivity (*a*), environmental stability (*b*), the proportion of neighbouring societies sharing the same strategy (*c*) and levels of political complexity (*d*,*e*). Yellow depicts foraging, blue plant-based agriculture and red animal husbandry. Political complexity levels: low – no jurisdictional authority beyond local communities, high – chiefdoms and states. The scale of the *Y*-axis changes between lower and upper boxes in (*a*,*b*) to account for low predicted probabilities of animal husbandry across all conditions. See Material and methods for details on sample and statistics.

The role that different environmental, social and historical factors have played in shaping subsistence patterns has been contested for decades in many disciplines (see discussion below, figure 2, table 1). Long-standing debates also persist regarding whether a limited set of factors can explain the variation in subsistence strategies across the globe [15,27,46,47]. Some scholars argue that in each case subsistence strategies reflect particular historical narratives defined by unique local conditions and events [15,27]. Others contend that a limited set of factors shape global patterns of subsistence [15,47]. Here we test these competing hypotheses by examining the degree to which environmental, social and historical variables alone or in combination can explain the variation in subsistence strategies in a global dataset.

**Table 1.** Hypothesized effects of factors influencing subsistence strategies. References intended as examples and not as a comprehensive review of the literature.

hypothesized factors	proposed effect with increased levels of factor	proposed effect with decreased levels of factor	no effect of factor
environmental productivity	associated with plant-based agriculture and animal husbandry [18–21] associated with decreased likelihood of foraging and animal husbandry [18,22–26]	associated with adoption of plant-based agriculture and animal husbandry [15,18,22,27] associated with increased likelihood of foraging and animal husbandry and decreased likelihood of plant-based agriculture [18,21–26] extreme low productivity associated with foraging due to limits on plant-based agriculture and animal husbandry [19,23,28] associated with increased likelihood of animal husbandry instead of foraging or plant-based agriculture [29]	no significant relationship [30]
environmental stability	associated with persistence of foraging instead of adoption of animal husbandry [22] associated with plant-based agriculture [19]	associated with animal husbandry [19,22–24,31,32], but with limits in extreme conditions [23] associated with increased likelihood of animal husbandry instead of foraging or plant-based agriculture [19,22,26]	—
varied topography	associated with advantage to pastoralists due to variation in resource availability [23]	associated with increased plant-based agriculture production [33,34] associated with limits on pastoralist expansion [30]	no consistent association for plant-based agriculture [33]
political complexity	associated with plant-based agriculture [35–40] associated with animal husbandry [38,39]	associated with foraging [35,38,39]	no consistent association [35–37,39,41]
related societies	more likely to have same subsistence strategy as closely related societies [39,42]	—	cultural features not derived from parent groups via phylogenesis, but from many groups via ethnogenesis [43]
contact with neighbouring societies	horizontal transmission of subsistence strategies leads to more similarity in subsistence among neighbouring societies [42,44]	preference to live near groups using different subsistence strategy for mutually beneficial trade purposes [30,45]	—

Methodological limitations of prior studies have hampered the resolution of these debates (table 2), partly because the evidence raised has often focused on specific case studies, relied on narrative arguments, or used qualitative methods for comparisons. These approaches produce detailed understanding at specific localities but do not provide quantitative evidence to test specific hypotheses regarding global-scale patterns. Other studies have taken an explicitly comparative and quantitative approach (table 2), but have often relied on relatively small samples, which can limit statistical power. Quantitative studies of subsistence patterns must also confront potentially confounding effects, including multi-collinearity within explanatory variables, as well as spatial and phylogenetic autocorrelation—all of which can lead to biases and increase the potential for reporting spurious effects (see Material and methods). Multiple factors probably shape subsistence strategies. Therefore, research must move beyond single-factor correlative studies and pursue multivariate models that can compare the relative influence of the wide variety of explanatory variables proposed in the literature (see discussion below and table 1). These methodological challenges have led to recent calls for more systematic and theory-driven hypothesis testing [47]. Recent research in the field of biogeography has overcome many of these methodological challenges through the use of model comparison techniques [53]. Multi-model inference approaches allow researchers to simultaneously compare multiple working hypotheses in a parsimonious process of model selection that seeks to balance model fit with model complexity [53,54]. Here we use this multi-model inference approach to evaluate the strength of evidence in support of a global model of dominant human subsistence strategies and to test competing hypotheses about the relative explanatory power of various drivers of subsistence strategy (see Material and methods for details).

**Table 2.** Addressing challenges in prior methodological approaches to examining the geography of subsistence.

limitation of prior methodological approach	alternative approach in the current study
small sample sizes limit statistical power (e.g. [30,48,49])	*n* = 818 societies spread across the globe
not accounting for spatial autocorrelation (e.g. [48–50])	included a neighbour effect (i.e. the proportion of 10 nearest neighbouring societies that share a society's subsistence strategy) as a predictor, and tested for unaccounted sources of spatial autocorrelation in model residuals (based on [4,51])
not accounting for phylogenetic autocorrelation (i.e. Galton's problem) (e.g. [48–50])	include random effect for the language family (based on [4,52])
testing a limited set of hypothesized factors and lack of model comparison, including studies with explanatory variables that are only environmental (e.g. [30,50]) or social (e.g. [48])	multi-model inference approach tests the strengths of individual hypotheses and all hypothesis combinations (based on [53])
qualitative assessment of selected case studies (e.g. [22])	multi-model inference approach with large global dataset allows for quantitative testing of multiple hypotheses [53]

## 2. Material and methods

We acquired all data from D-PLACE (Database of Places, Language, Culture and Environment [17,51,55,56]). We only used data collected in a relatively narrow time span (1860–1960) to avoid the effects of changing environmental and social conditions, including long-term transitions in subsistence strategies, as well as the possibility that over the course of human history multiple groups may have occupied a given location. We used variables describing subsistence economy (EA001—EA005) to determine the dominant subsistence strategy, which we defined as the strategy relied on for more than 56% of total subsistence. These variables are all categorical (ordinal), ranging from category 0 (0–5% dependence) to category 9 (86–100% dependence). We used the 56% figure to define the dominant subsistence strategy as category 6 ranges from 56 to 65% dependence, meaning that the majority of subsistence was obtained from this strategy, whereas category 5 (46–55% dependence) includes instances in which less than a majority (less than 50%) of dependence was from the given subsistence strategy. We summed the hunting (EA002), gathering (EA001) and fishing (EA003) categories to represent dependence on the foraging subsistence strategy. We omitted from our analyses societies for which multiple strategies (foraging, animal husbandry, or plant-based agriculture) contributed equally, with no one strategy contributing more than 56%. This protocol resulted in the following distribution of dominant subsistence strategies: foraging (i.e. hunting + gathering + fishing): 270 societies, plant-based agriculture: 504 societies, animal husbandry: 44 societies (figure 1). We also concluded that our results were qualitatively similar when we altered the definition of animal husbandry to be inclusive of societies that depended in part on animal husbandry. We recorded data on political complexity (EA033) at two levels (1 = no jurisdictional authority beyond local communities, 2 = chiefdoms and states). We emphasize that our analysis focuses on identifying factors shaping dominant subsistence strategy, which is distinct from subsistence diversity. For example, some societies that are primarily (greater than 56%) plant-based agriculturalists may still obtain a substantial amount of food from foraging activities, whereas other societies may rely almost exclusively on plant-based agriculture. Variation also exists within the three subsistence categories. For example, the ecological, social and historical factors that shape the degree to which a society hunts may be different to those that influence the degree to which the group relies on fishing or gathering of food resources. We suggest that future studies can explore factors that influence diversity within and among subsistence categories, and how these factors may differ from those we identify here as shaping dominant subsistence strategy.

Climate data were from the baseline historical (1900–1949) CCSM ecoClimate model [57]. This time focus matches the studied time frame of the majority of the societies in our dataset [51]. We also note that substantial climate change did not occur between 1860 and 1900, the time frame during which data on the remaining societies were collected [58]. We derived topographic data (slope and elevation) from the Global Multi-resolution Terrain Elevation Data 2010 [59]. We extracted all climatic and topographic variables for the localities of the societies in our sample based on global maps at a 0.5 × 0.5° resolution.

We tested for an effect of environment on dominant subsistence strategy by characterizing the mean, variance and predictability of temperature and precipitation within years, as well as the average local net primary productivity at the localities of our cultural samples. To avoid multi-collinearity in our explanatory models, we reduced these often highly correlated environmental predictors to orthogonal components via principal components analysis. First, we transformed variables to meet assumptions of normality (when needed), centred and scaled. The Kaiser rule and parallel analysis [60] informed the number of factors retained for analysis. The PCA produced three main composite variables: (i) ‘environmental stability’ describes a gradient of increasing mean temperature, temperature predictability, mean precipitation, lower precipitation variance and decreasing temperature variance; (ii) ‘topographic complexity’ describes a gradient of increasing slope and elevation and (iii) ‘environmental productivity’ describes a gradient of increasing mean precipitation, lower precipitation variance, precipitation predictability and net primary productivity (results in the electronic supplementary material, table S1). To capture the potential effects of horizontal transmission, we included as a predictor the proportion of the 10 nearest neighbouring societies that share a society's subsistence strategy (neighbour effect). When we varied the number of neighbours used in the analysis (e.g. 5, 10, 15 or 20 nearest neighbours) results remained consistent. Horizontal transmission requires societies to exist concurrently, which we assumed to be true given that the data analysed represent a relatively short time span.

Cross-cultural comparative analyses must also grapple with the non-independence of societies that share a common cultural background, also referred to as phylogenetic autocorrelation or Galton's problem [61,62]. One approach to the problem involved the development of the standard cross-cultural sample (SCCS), which includes one representative from each set of theoretically independent clusters [63]. Critics of this approach note that societies in the sample will be related at some point in history, and that the degree of relatedness, and thus non-independence, will vary across the sample [64]. Recent analyses have demonstrated that the SCCS does not remove the effects of phylogenetic autocorrelation for many variables, including those covering subsistence activities [61]. Others argue for the use of phylogenetic approaches to identify independent instances of cultural change [42]. However, the current lack of a robust global cultural phylogeny prohibits such an approach. On a global scale, language families represent the most reliable data on the historical relationships among societies [65]. Based on methods used in previous studies in evolution [52] and in cross-cultural analyses [4], we included language family as a random effect in our models to account for historical relatedness of societies and to test for the potential role of vertical transmission of subsistence strategies.

In order to assess the relative influence of all predictor variables and test previous hypotheses, we implemented a multi-model inference approach [53,54]. This multi-model inference approach allowed us to estimate the relative importance and most likely effect sizes of different putative predictors while simultaneously accounting for model uncertainty. We ran the multinomial mixed models using the Glimmix procedure in SAS University Edition. We only included societies in the analyses for which we could obtain complete subsistence, environmental, topographical, geographical and political complexity data (see electronic supplementary material). We ran all possible models based on the alternative combinations of predictors in our candidate set and calculated the Akaike information criterion corrected for small samples (AICc) for each model, and subsequently the change in AIC (ΔAICc) relative to the best-supported model (i.e. the model with the lowest AICc), and the Akaike weight (AICw), which is interpreted as the conditional probability of the model [53]. As no model parametrization was clearly the best (i.e. AICw ≥ 0.9, table 3), we computed model average parameters by averaging across all models (see [53]). This average model offers unbiased estimates of the magnitude and direction of the effect of each predictor. We used model weights to estimate the relative importance of each predictor, which is defined as the weight of the evidence in favour of including a particular predictor in our statistical model [53]. We report the results of both the average model (table 4) and the best-supported model (electronic supplementary material, table S2).

**Table 3.** Support for alternative models of dominant subsistence strategy. Only the five best-supported models are shown. All models include intercept and a random effect for language family. AICc refers to small-sample Akaike Information Criterion, ΔAICc is the change in AICc relative to the best-supported model (i.e. model with lowest AICc), and AICw is Akaike weight or the conditional probability of a model.

model	AICc	ΔAICc	AICw
productivity + stability + politics + neighbour effect	512.20	0.00	0.74
productivity + stability + topography + politics + neighbour effect	514.41	2.21	0.24
productivity + politics + neighbour effect	520.87	8.67	0.009
productivity + topography + politics + neighbour effect	521.62	9.42	0.007
productivity + stability + neighbour effect	539.66	27.46	<0.001

**Table 4.** Multi-model average for models of dominant subsistence strategy. *N* = 818 societies. Political complexity coded as 2 levels (low = no jurisdictional authority beyond local communities, high = chiefdoms and states). RVI is calculated as the sum of AIC weights for all models containing the explanatory variable. Foraging serves as the reference category.

parameter	level	β-coefficient	s.e.	RVI
intercept	animal husbandry	−8.71	2.97	1
	plant-based agriculture	−5.92	0.90	
productivity	animal husbandry	−2.55	0.72	1
	plant-based agriculture	0.06	0.24	
stability	animal husbandry	−0.49	0.71	0.98
	plant-based agriculture	1.03	0.32	
topography	animal husbandry	0.09	0.24	0.25
	plant-based agriculture	0.08	0.12	
politics	animal husbandry	5.17	1.48	1
	plant-based agriculture	1.72	0.43	
neighbour effect	animal husbandry	−12.37	3.84	1
	plant-based agriculture	4.02	0.67	
$R_{\mathrm{GLMM}}^{2}$	0.86			

We calculated $R_{\mathrm{GLMM}}^{2}$ values for all possible models (following [66]) and subsequently the weighted average model. Owing to the differences in calculating *R*^2^ for mixed models and linear models, we only calculated the multi-model average $R_{\mathrm{GLMM}}^{2}$ from the subset of mixed models including the random effect of the language family. Multi-model average coefficients from the mixed models (*n* = 32) and across all models (*n* = 64) are identical (table 4; electronic supplementary material, table S3). The Moran's I correlogram (electronic supplementary material, figure S1) indicates that there is no evidence of any unaccounted sources of spatial autocorrelation in our best-supported model.

## 3. Results and discussion

Three hypotheses propose that environmental productivity and stability place constraints on subsistence strategies (table 1, figure 2). First, some researchers argue that greater environmental productivity and stability are critical prerequisites for the development of plant-based agriculture and animal husbandry [18–20]. This hypothesis therefore predicts that food production focused on plant-based agriculture or animal husbandry should be more likely in more environmentally productive and stable locations. A second hypothesis claims that low levels of environmental productivity and stability may favour the adoption of plant-based agriculture or animal husbandry [15,18,22,27]. A third set of researchers argue that plant-based agriculture has displaced other forms of subsistence in all but the most marginal environments, where extreme and variable temperatures and precipitation, or short growing seasons, require more mobile subsistence in the forms of foraging and animal husbandry [18,22–24,30]. This hypothesis predicts that foraging and animal husbandry should be less likely to occur in environmentally productive and stable locations.

In our analyses, both environmental productivity and stability have important effects on dominant subsistence strategies (tables 3 and 4). We find support for the hypothesis that animal husbandry is more likely to be a dominant subsistence strategy in environmentally less productive regions (figure 2*a*, and a negative β-coefficient (−2.55), indicating animal husbandry is associated with less productive environments than foraging, which serves as the reference category in the analysis). However, we did not find a significant effect of environmental productivity on the likelihood of plant-based agriculture versus foraging (table 2, s.e. of the β-coefficient for plant-based agriculture bounds zero). These results are similar to those from a previous global-sample study that found net primary productivity did not vary significantly across lands inhabited by foraging versus plant-based agricultural societies [30]. We also note that different species of domesticated plants and animals have different environmental requirements, and these differences are not captured in our current analysis, which may account for some of the remaining unexplained variations in dominant subsistence strategies.

In addition, although some have argued that pastoralism is well suited to environmental instability [19,22,24], our analyses show no significant effect of environmental stability on the probability of animal husbandry being a dominant subsistence strategy (table 4, electronic supplementary material, table S2). In the most stable environments, the predicted probability of plant-based agriculture as a dominant subsistence strategy increases to greater than 80% (figure 2*b*). Plant-based agriculture is a less mobile subsistence strategy than foraging or animal husbandry, and therefore plant-based agricultural communities may be more susceptible to the risks associated with unpredictable environmental conditions.

Researchers also debate the potential effects topography has on subsistence strategies. However, previous research is inconclusive, with some case studies finding no influence of topography and others concluding that topographic complexity has a substantial negative effect on important subsistence variables [67,68]. Our global-scale analysis finds that topography is neither a robust nor a significant predictor of dominant subsistence strategies. Specifically, the term is absent in the best-supported model (ΔAICc < 2), and the relative variable importance of topography (RVI = 0.25) is substantially less than that of any other predictor (all other RVI = 1; table 4).

Previous studies have also debated the relationship between political complexity and the distribution of different subsistence strategies (table 1, figure 2). One dominant view in anthropological discourse depicts foraging societies as non-stratified, politically simple, autonomous bands and associates plant-based agriculture with increasing political complexity [35,36,40]. Some argue that plant-based agriculture allows for the production of surplus food, which supports social stratification and political complexity [11]. Others note that more complex political systems can improve agricultural efficiency through a variety of mechanisms including property rights and water distribution systems [36]. However, other researchers point to multiple archaeological case studies to argue that political complexity and subsistence strategies may not be so tightly linked. For example, multiple foraging societies developed chiefdoms in the absence of plant-based agriculture [35,37]; some societies with only local levels of the political organization have developed intensive forms of plant-based agriculture and produced agricultural surplus [36,41] and state control can often reduce the efficiency of agricultural production [36,69]. Although exceptional cases clearly exist, our comparative analysis finds evidence for a global pattern in which political complexity is linked to dominant subsistence strategy (tables 3 and 4, figure 2*d,e*). For example, societies with higher levels of political complexity, in the form of chiefdoms and states, are greater than 50% more likely to use a plant-based agriculture as their dominant subsistence strategy.

Scholars also debate the degree to which vertical and horizontal transmission of information affect different cultural traits, including subsistence strategies [42]. The vertical transmission of subsistence strategies from one generation to another would lead to similarities in subsistence across closely related contemporary societies. To approximate this potential effect, we included language family in our analysis (see Material and methods) and found that this predictor is indeed significantly associated with a society's dominant subsistence strategy (table 3, electronic supplementary material, table S3). Similarly, we used neighbouring groups in order to estimate the potential effects of horizontal transmission. Neighbouring societies may exhibit similar subsistence strategies due to the horizontal transmission of ideas and technologies, and due to experiencing similar environments. Alternatively, neighbours may be less similar than expected by chance if living near groups with different subsistence strategies maximizes opportunities for mutually beneficial trade, a frequent explanation for why societies practising animal husbandry and foraging often neighbour plant-based agricultural groups [30,45]. To test these hypotheses, we included the proportion of neighbouring societies sharing the same subsistence strategies into our analysis. The dominant subsistence strategies of neighbouring societies vary: plant-based agricultural groups tend to cluster spatially with other plant-based agricultural groups, foraging societies maintain a moderate level of spatial clustering and societies practising animal husbandry cluster the least (table 4, figures 1 and 2*c*). Such variation in spatial clustering may be due to either different degrees of horizontal transmission across regions, or due to different histories. For example, where closely related plant-based agricultural groups occupy continuous geographical areas, horizontal transmission of agriculture could result in spatial signatures in our data similar to those produced by vertical transmission of agricultural practices. Despite these limitations on untwining transmission histories, we find informative differences in the spatial patterns exhibited by the three dominant subsistence strategies.

Before 11 000 years ago, all human societies foraged for food, but more recently plant-based agriculture or animal husbandry became the dominant subsistence strategy for most human groups. By applying a multi-model inference approach that is now widely used in biogeography, we have been able to provide quantitative estimates of the degree to which different environmental, social and historical conditions have shaped the dominant subsistence strategies used in recent times. We conclude that some hypotheses are supported whereas others are not (figure 2), and that none of the putative environmental, social and historical predictors suggested to date is individually capable of predicting the entire range of global variation we observe in dominant subsistence strategies across the globe (ΔAICc > 205 for models with only individual factors). Instead, the best-supported model (ΔAICc < 2; table 3) includes multiple environmental, social and historical factors. This limited suite of variables jointly describes the vast majority of the variation in dominant subsistence strategy across the world $\left(R_{\mathrm{GLMM}}^{2}=0.86\right)$. Ultimately, subsistence strategies are driven by human agency and individual human decisions [70]; and our results imply a role for various environmental, social and historical factors in shaping these subsistence decisions. Although some researchers have argued that unique local conditions and events shape subsistence strategies [15,27,46,47], our results support assertions [47] that global patterns exist in which environmental conditions, political complexity and the strategies used by nearby and closely related groups are all linked to dominant subsistence strategies.

## Supplementary Material

Supplementary Table and Figure

## Data accessibility

The full dataset used in our analyses is available via Dryad Digital Repository: http://dx.doi.org/10.5061/dryad.884r935 [71].
