# Supplementary material for: The global geography of human subsistence
Source: R Soc Open Sci. 2018 Sep 26;5(9):171897. doi: 10.1098/rsos.171897 (PMC6170550; doi:10.1098/rsos.171897)
Supplement: Supplementary Table and Figure [file rsos171897supp1.docx]

**Supplementary Information**

**Supplementary Table 1.** Principal components analysis of ecological variables, with varimax rotation and main contributors in bold.

| variable | climatic stability (PC1) |  | environmental productivity (PC3) |  | topographic complexity (PC2) |  | uniqueness |
| --- | --- | --- | --- | --- | --- | --- | --- |
|  |  |  |  |  |  |  |  |
| sqrt (annual mean temperature) | **0.86** |  | -0.09 |  | -0.42 |  | 0.08 |
| ln (annual temperature variance) | **-0.84** |  | -0.43 |  | 0.14 |  | 0.09 |
| Temperature predictability | **0.90** |  | 0.31 |  | -0.13 |  | 0.08 |
|  |  |  |  |  |  |  |  |
| sqrt (annual mean precipitation) | **0.50** |  | **0.80** |  | 0.08 |  | 0.11 |
| ln (annual precipitation variance) | **0.64** |  | **0.56** |  | 0.18 |  | 0.24 |
|  |  |  |  |  |  |  |  |
| Precipitation predictability | -0.03 |  | **0.88** |  | -0.23 |  | 0.17 |
|  |  |  |  |  |  |  |  |
| sqrt (net primary productivity) | 0.43 |  | **0.73** |  | 0.16 |  | 0.26 |
|  |  |  |  |  |  |  |  |
| sqrt (elevation) | -0.12 |  | -0.14 |  | **0.86** |  | 0.23 |
| ln (slope) | -0.13 |  | 0.11 |  | **0.90** |  | 0.17 |
| SS loadings | 3.14 |  | 2.57 |  | 1.87 |  |  |
| Cumulative variance | 0.35 |  | 0.63 |  | 0.84 |  |  |

**Supplementary Table 2.** Results from best-supported model of dominant subsistence mode.

| Parameter | Level | β coefficients | Standard errors | p*-*values |
| --- | --- | --- | --- | --- |
| Intercept | Animal husbandry | -8.73 | 2.99 | 0.004 |
|  | Plant-based agriculture | -5.94 | 0.90 | <.0001 |
|  |  |  |  |  |
| Productivity | Animal husbandry | -2.54 | 0.72 | 0.0005 |
|  | Plant-based agriculture | 0.07 | 0.23 | 0.78 |
|  |  |  |  |  |
| Stability | Animal husbandry | -0.48 | 0.71 | 0.50 |
|  | Plant-based agriculture | 1.06 | 0.38 | 0.0009 |
|  |  |  |  |  |
| Politics | Animal husbandry | 5.15 | 1.48 | 0.0005 |
|  | Plant-based agriculture | 1.72 | 0.43 | <.0001 |
|  |  |  |  |  |
| Neighbor effect | Animal husbandry | -12.36 | 3.86 | 0.002 |
|  | Plant-based agriculture | 4.06 | 0.67 | <.0001 |
|  |  |  |  |  |
| **R^2^_GLMM_** | **0.86** |  |  |  |

**Supplementary Table 3**. Model averaged parameters across all 64 possible candidate models with and without language family as a random effect.

| parameter | level | β coefficient | standard error | relative variable importance (RVI) |
| --- | --- | --- | --- | --- |
|  |  |  |  |  |
| Intercept | Animal husbandry | -8.71 | 2.97 | 1.00 |
|  | Plant-based agriculture | -5.92 | 0.90 |  |
|  |  |  |  |  |
| Productivity | Animal husbandry | -2.55 | 0.72 | 1.00 |
|  | Plant-based agriculture | 0.06 | 0.24 |  |
|  |  |  |  |  |
| Stability | Animal husbandry | -0.49 | 0.71 | 0.98 |
|  | Plant-based agriculture | 1.03 | 0.32 |  |
|  |  |  |  |  |
| Topography | Animal husbandry | 0.09 | 0.24 | 0.25 |
|  | Plant-based agriculture | 0.08 | 0.112 |  |
|  |  |  |  |  |
| Politics | Animal husbandry | 5.17 | 1.48 | 1.00 |
|  | Plant-based agriculture | 1.72 | 0.43 |  |
|  |  |  |  |  |
| Neighbor effect | Animal husbandry | -12.37 | 3.834 | 1.00 |
|  | Plant-based agriculture | 4.02 | 0.67 |  |
|  |  |  |  |  |
| Language family |  |  |  | 1.00 |

**Supplementary Table 4.** Societies included in the analysis of dominant subsistence type. n = 818 societies (foraging = 270; plant-based agriculture = 504; animal husbandry = 44).

| society name  (based on ethnographic atlas) | ethnographic atlas ID | latitude | longitude | dominant subsistence type |
| --- | --- | --- | --- | --- |
| Pedi | Ab15 | -25 | 30 | Plant-based agriculture |
| Barea | Ca32 | 16 | 38 | Plant-based agriculture |
| Kunama | Ca33 | 15 | 37 | Plant-based agriculture |
| Beni Amer | Ca36 | 18 | 38 | Animal husbandry |
| Tigre | Ca38 | 17 | 39 | Animal husbandry |
| Ajie | Ih5 | -21.37 | 165.47 | Plant-based agriculture |
| Easter Island | Ij9 | -27.12 | -109.36 | Plant-based agriculture |
| Satudene | Na16 | 65 | -119 | Foraging |
| Dogrib | Na15 | 63 | -117 | Foraging |
| Slave | Na17 | 61 | -120 | Foraging |
| Hupa | Nb35 | 41 | -123 | Foraging |
| Mattole | Nb38 | 40.35 | -123.47 | Foraging |
| Makah | Nb24 | 48.33 | -124.62 | Foraging |
| Lake Miwok | Nc21 | 38.75 | -123.2 | Foraging |
| Wintu | Nc14 | 41 | -122 | Foraging |
| Lake Yokuts | Nc24 | 36 | -120 | Foraging |
| Wishram | Nd18 | 46 | -121 | Foraging |
| Takelma | Nb30 | 42 | -123 | Foraging |
| Klamath | Nc8 | 43 | -122 | Foraging |
| Modoc | Nc9 | 42 | -122 | Foraging |
| Umatilla | Nd19 | 46 | -119 | Foraging |
| Alsea | Nb28 | 44 | -124 | Foraging |
| Siuslaw | Nb29 | 44 | -124 | Foraging |
| Lassik | Nb37 | 40 | -123 | Foraging |
| Wappo | Nc20 | 38.22 | -122.56 | Foraging |
| Coeur d’Alene | Nd14 | 48 | -117 | Foraging |
| Flathead | Nd12 | 46 | -113 | Foraging |
| Kalispel | Nd13 | 49 | -117 | Foraging |
| Sinkyone | Nb39 | 39.73 | -123.6 | Foraging |
| Klallam | Nb16 | 48 | -123 | Foraging |
| Lillooet | Nd9 | 50.02 | -124.78 | Foraging |
| Quinault | Nb25 | 47.42 | -124.16 | Foraging |
| Coast Yuki | Nc15 | 39.34 | -123.77 | Foraging |
| Thompson | Nd10 | 50 | -122 | Foraging |
| Arapaho | Ne9 | 40 | -103 | Foraging |
| Huchnom | Nc16 | 39 | -123 | Foraging |
| Cheyenne | Ne5 | 39 | -104 | Foraging |
| Chippewa | Na36 | 49 | -96 | Foraging |
| Wukchumni | Nc25 | 36 | -119 | Foraging |
| Wiyot | Nb36 | 39.81 | -123.55 | Foraging |
| Hidatsa | Ne15 | 47 | -101 | Plant-based agriculture |
| Chimariko | Nb33 | 41 | -123 | Foraging |
| Karok | Nb34 | 42 | -123 | Foraging |
| Eastern Pomo | Nc18 | 39 | -123 | Foraging |
| Northern Pomo | Nc17 | 39 | -123 | Foraging |
| Southern Pomo | Nc19 | 38.55 | -122.94 | Foraging |
| Achomawi | Nc10 | 41 | -121 | Foraging |
| Atsugewi | Nc4 | 41 | -121 | Foraging |
| Shasta | Nb32 | 41 | -122 | Foraging |
| Kamia | Nh20 | 33 | -115 | Foraging |
| Yana | Nc11 | 41 | -122 | Foraging |
| Agaiduka | Nd46 | 44 | -112 | Foraging |
| Antarianunts | Nd49 | 37 | -110 | Foraging |
| Bannock | Nd63 | 43 | -112 | Foraging |
| Bohogue | Nd45 | 43 | -112 | Foraging |
| Chemehuevi | Nd54 | 35 | -115 | Foraging |
| Gosiute | Nd48 | 40 | -114 | Foraging |
| Kaibab | Nd53 | 36 | -113 | Foraging |
| Kawaiisu | Nc27 | 35 | -118 | Foraging |
| Kuyuidokado | Nd27 | 40 | -119 | Foraging |
| Las Vegas | Nd55 | 36 | -115 | Foraging |
| Luiseno | Nc33 | 33 | -117 | Foraging |
| Moache | Nd60 | 37 | -105 | Foraging |
| Moanunts | Nd59 | 39 | -112 | Foraging |
| Moapa | Nd51 | 36 | -115 | Foraging |
| Pahvant | Nd57 | 39 | -113 | Foraging |
| Panguitch | Nd50 | 38 | -112 | Foraging |
| San Juan | Nd56 | 36 | -111 | Foraging |
| Shivwits | Nd52 | 36 | -117 | Foraging |
| Southern Ute | Nd2 | 38 | -109 | Foraging |
| Taviwatsiu | Nd61 | 40 | -105 | Foraging |
| Uintah | Nd58 | 40 | -112 | Foraging |
| Uncompahgre | Nd62 | 39 | -107 | Foraging |
| White Knife | Nd43 | 41 | -117 | Foraging |
| Wind River | Nd64 | 43 | -109 | Foraging |
| Mataco | Sh1 | -24 | -63 | Foraging |
| Coos | Nb21 | 43 | -124 | Foraging |
| Kiowa | Ne17 | 36 | -99 | Foraging |
| Alkatcho | Nb10 | 53 | -126 | Foraging |
| Mpongwe | Ae46 | -2 | 10 | Plant-based agriculture |
| Kanuri | Cb19 | 12 | 13 | Plant-based agriculture |
| Hamyan | Cd14 | 35 | 2 | Animal husbandry |
| Koiari | Ie24 | -9 | 148 | Plant-based agriculture |
| Chakma | Ei10 | 23 | 92 | Plant-based agriculture |
| Dard | Ee5 | 35 | 73 | Plant-based agriculture |
| Tahltan | Na27 | 58 | -131 | Foraging |
| Tanaina | Na26 | 62 | -144 | Foraging |
| Tolowa | Nb6 | 42 | -124 | Foraging |
| Tututni | Nb31 | 42 | -124 | Foraging |
| Jicarilla | Nh16 | 36 | -104 | Foraging |
| Kiowa Apache | Ne2 | 36 | -99 | Foraging |
| Mescalero | Nh15 | 33 | -103 | Foraging |
| Western Apache | Nh17 | 34 | -110 | Foraging |
| Klikitat | Nd17 | 46 | -122 | Foraging |
| Puyallup | Nb17 | 47 | -122 | Foraging |
| Sanpoil | Nd4 | 48 | -119 | Foraging |
| Tillamok | Nb20 | 44.75 | -123.72 | Foraging |
| Wenatchi | Nd16 | 48 | -121 | Foraging |
| Eastern Ojibwa | Na39 | 46 | -85 | Foraging |
| Walapai | Nd65 | 36 | -114 | Foraging |
| Menomini | Nf9 | 46 | -88 | Foraging |
| Tolkepaya | Nd67 | 34 | -114 | Foraging |
| Northern Saulteaux | Na33 | 52 | -98 | Foraging |
| Assiniboin | Ne11 | 48 | -106 | Foraging |
| Crow | Ne4 | 45 | -108 | Foraging |
| Oto | Nf11 | 40 | -95 | Foraging |
| Teton | Ne8 | 43 | -103 | Foraging |
| Kewyipaya | Nh23 | 34 | -111 | Foraging |
| Yavapai | Nd66 | 35 | -112 | Foraging |
| Yaqui | Ni7 | 28 | -110 | Plant-based agriculture |
| Atsakudokwa | Nd23 | 42 | -118 | Foraging |
| Beatty | Nd33 | 37 | -116 | Foraging |
| Cahuilla | Nc31 | 33 | -116 | Foraging |
| Comanche | Ne3 | 33 | -100 | Foraging |
| Cupeno | Nc32 | 33 | -117 | Foraging |
| Eastern Mono | Nd30 | 37 | -118 | Foraging |
| Elko Shoshoni | Nd42 | 41 | -116 | Foraging |
| Ely Shoshoni | Nd38 | 39 | -115 | Foraging |
| Hamilton | Nd37 | 39 | -116 | Foraging |
| Hukundika | Nd5 | 42 | -112 | Foraging |
| Kidutokado | Nd24 | 42 | -120 | Foraging |
| Lida Shoshoni | Nd34 | 37 | -117 | Foraging |
| Mahaguaduka | Nd35 | 39 | -117 | Foraging |
| Monachi | Nc23 | 37 | -119 | Foraging |
| Sawakudokwa | Nd25 | 41 | -118 | Foraging |
| Serrano | Nc30 | 34 | -117 | Foraging |
| Spring Valley | Nd39 | 40 | -115 | Foraging |
| Tagotoka | Nd21 | 43 | -117 | Foraging |
| Toedokado | Nd28 | 40 | -118 | Foraging |
| Tubaduka | Nd40 | 40 | -113 | Foraging |
| Tunava | Nd29 | 37 | -118 | Foraging |
| Wadadokado | Nd22 | 43 | -119 | Foraging |
| Wadaduka | Nd41 | 41 | -116 | Foraging |
| Wadatkuht | Nd26 | 41 | -120 | Foraging |
| Wiyambituka | Nd36 | 39 | -117 | Foraging |
| Tehuelche | Sg4 | -46 | -70 | Foraging |
| Andamese | Eh1 | 12 | 93 | Foraging |
| Yahgan | Sg1 | -55.02 | -68.98 | Foraging |
| Ganda | Ad7 | 1 | 32 | Plant-based agriculture |
| Kwere | Ad27 | -7 | 39 | Plant-based agriculture |
| Swazi | Ab2 | -27 | 32 | Plant-based agriculture |
| Teke | Ac19 | -3 | 15 | Plant-based agriculture |
| Zigula | Ad28 | -5.84 | 38.79 | Plant-based agriculture |
| Afar | Ca6 | 12 | 42 | Animal husbandry |
| Esa | Ca10 | 10 | 43 | Animal husbandry |
| Gibe | Ca12 | 8 | 37 | Plant-based agriculture |
| Mogh | Ei9 | 20.49 | 92.64 | Plant-based agriculture |
| Tamil | Eg2 | 11 | 79 | Plant-based agriculture |
| Abkhaz | Ci12 | 43 | 41 | Animal husbandry |
| Ob Ostyak | Ec10 | 62 | 74 | Foraging |
| Baffinland | Na13 | 65.24 | -64.63 | Foraging |
| Polar Inuit | Na14 | 78 | -70 | Foraging |
| Tareumiut | Na2 | 71 | -157 | Foraging |
| Angmagsalik | Na24 | 66 | -37 | Foraging |
| Carrier | Na19 | 54 | -124 | Foraging |
| Chipewyan | Na30 | 60 | -105 | Foraging |
| Ingalik | Na8 | 62 | -160 | Foraging |
| Kutchin | Na20 | 66 | -135 | Foraging |
| Sarsi | Ne7 | 54 | -110 | Foraging |
| Sekani | Na28 | 56 | -123 | Foraging |
| Chiricahua | Nh1 | 31 | -108 | Foraging |
| Lipan | Nh24 | 30 | -100 | Foraging |
| Bellabella | Nb23 | 52 | -128 | Foraging |
| Nootka | Nb11 | 49.16 | -125.85 | Foraging |
| Klahuse | Nb12 | 51 | -125 | Foraging |
| Stalo | Nb27 | 49 | -122 | Foraging |
| Bellacoola | Nb9 | 52 | -127 | Foraging |
| Comox | Nb14 | 49.72 | -124.97 | Foraging |
| Cowichan | Nb26 | 49.01 | -122.73 | Foraging |
| Lummi | Nb15 | 48.84 | -123.27 | Foraging |
| Haisia | Nb8 | 54 | -129 | Foraging |
| Squamish | Nb13 | 50 | -123 | Foraging |
| Sinkaitk | Nd15 | 49 | -120 | Foraging |
| Gros Ventre | Ne1 | 48.78 | -108.98 | Foraging |
| Montagnais | Na32 | 48 | -72 | Foraging |
| Chilcotin | Nd8 | 52 | -122 | Foraging |
| Rainy River | Na37 | 49 | -92 | Foraging |
| Quileute | Nb18 | 47.91 | -124.59 | Foraging |
| Kiliwa | Nc34 | 31 | -115 | Foraging |
| Mapuche | Sg2 | -39 | -68 | Plant-based agriculture |
| Ona | Sg3 | -54 | -69 | Foraging |
| Botocudo | Sj5 | -18 | -42 | Foraging |
| Fur | Cb17 | 12 | 24 | Plant-based agriculture |
| Kutenai | Nd7 | 50 | -117 | Foraging |
| Tlingit | Nb22 | 58.12 | -133.92 | Foraging |
| Tsimshian | Nb7 | 54.64 | -130.25 | Foraging |
| Ungazikmit | ec14 | 64 | 173 | Foraging |
| Mansi | ec15 | 63 | 62 | Foraging |
| Latvians | ch21 | 57 | 24 | Plant-based agriculture |
| Digo | Ad30 | -4 | 39 | Plant-based agriculture |
| Pare | Ad36 | -4 | 38 | Plant-based agriculture |
| Assini | Af40 | 5.27 | -3.58 | Plant-based agriculture |
| Abarambo | Ai27 | 4 | 27 | Plant-based agriculture |
| Basari | Ag48 | 9 | 1 | Plant-based agriculture |
| Attie | Af41 | 6 | -4 | Plant-based agriculture |
| Sara | Ai22 | 9 | 18 | Plant-based agriculture |
| Kabyle | Cd4 | 36 | 4 | Plant-based agriculture |
| Kafa | Ca30 | 7 | 36 | Plant-based agriculture |
| Mota | Ih1 | -13.84 | 167.7 | Plant-based agriculture |
| Rotumans | Ih6 | -12.5 | 177.07 | Plant-based agriculture |
| Nuri | Ea5 | 36 | 71 | Plant-based agriculture |
| Labrador Inuit | Na23 | 58 | -65 | Foraging |
| Kwakiutl | Nb3 | 51 | -127.49 | Foraging |
| Naskapi | Na5 | 58 | -70 | Foraging |
| Cochiti | Nh7 | 36 | -106 | Plant-based agriculture |
| Taos | Nh6 | 37 | -106 | Plant-based agriculture |
| Lengua | Sh9 | -23 | -59 | Foraging |
| Chamacoco | Sh6 | -20 | -59 | Foraging |
| Eyak | Nb5 | 60.34 | -144.85 | Foraging |
| Haida | Nb1 | 54 | -132 | Foraging |
| Evenk | ec16 | 55 | 112 | Foraging |
| Negidal | ec17 | 52 | 134 | Foraging |
| Ulch | ec18 | 50 | 136 | Foraging |
| Orok | ec19 | 54.01 | 142.71 | Foraging |
| Oroch | ec20 | 51 | 138 | Foraging |
| Udihe | ec21 | 47 | 136 | Foraging |
| Udmurt | ch26 | 57 | 53 | Plant-based agriculture |
| Besermyan | ch13 | 58 | 52 | Plant-based agriculture |
| Gagauz | ch17 | 45.44 | 29.32 | Plant-based agriculture |
| Erzia mordva | ch15 | 53 | 50 | Plant-based agriculture |
| Lithuanian Karaim | ch22 | 55 | 23 | Plant-based agriculture |
| Bemba | Ac3 | -11 | 31 | Plant-based agriculture |
| Boki | Af17 | 6 | 9 | Plant-based agriculture |
| Rumbi | Ae35 | 0 | 28 | Plant-based agriculture |
| Ekoi | Af18 | 6 | 8 | Plant-based agriculture |
| Giriama | Ad32 | -3 | 40 | Plant-based agriculture |
| Gisu | Ad9 | 1 | 34 | Plant-based agriculture |
| Herero | Ab1 | -21 | 16 | Animal husbandry |
| Iwa | Ad15 | -10 | 32 | Plant-based agriculture |
| Kaguru | Ad50 | -6 | 37 | Plant-based agriculture |
| Kongo | Ac14 | -7 | 15 | Plant-based agriculture |
| Kundu | Ae45 | 10 | 9 | Plant-based agriculture |
| Makonde | Ac41 | -11 | 40 | Plant-based agriculture |
| Pokomo | Ad33 | -1 | 40 | Plant-based agriculture |
| Poto | Ae29 | 2 | 22 | Foraging |
| Sanga | Ae43 | 2 | 16 | Plant-based agriculture |
| Sena | Ac40 | -18 | 35 | Plant-based agriculture |
| Songe | Ae18 | -6 | 25 | Plant-based agriculture |
| Songola | Ae11 | -4 | 26 | Foraging |
| Sumbwa | Ad47 | -4 | 32 | Plant-based agriculture |
| Venda | Ab6 | -23 | 30 | Plant-based agriculture |
| Mandja | Ai24 | 6 | 18 | Plant-based agriculture |
| Soninke | Ag25 | 15 | 10 | Plant-based agriculture |
| Mossi Ouagadou | Ag47 | 12 | -2 | Plant-based agriculture |
| Senufo | Ag32 | 10 | -6 | Plant-based agriculture |
| Abron | Af38 | 7.53 | -2.15 | Plant-based agriculture |
| Anyi | Af39 | 7 | -4 | Plant-based agriculture |
| Ashanti | Af3 | 7 | -2 | Plant-based agriculture |
| Bakwe | Af46 | 5 | -7 | Plant-based agriculture |
| Baule | Af9 | 8 | -5 | Plant-based agriculture |
| Buem | Af37 | 7.22 | 0.46 | Plant-based agriculture |
| Edo | Af24 | 6 | 6 | Plant-based agriculture |
| Ewe | Af36 | 7 | 1 | Plant-based agriculture |
| Fanti | Af42 | 6 | -1 | Plant-based agriculture |
| Kukuruku | Af26 | 7 | 6 | Plant-based agriculture |
| Vai | Af58 | 6.89 | -11.2 | Plant-based agriculture |
| Mangbetu | Ai11 | 3 | 28 | Plant-based agriculture |
| Kuku | Aj15 | 4 | 32 | Plant-based agriculture |
| Masai | Aj2 | -2 | 36 | Animal husbandry |
| Midobi | Cb11 | 16 | 27 | Animal husbandry |
| Tekna | Cd9 | 28 | -11 | Plant-based agriculture |
| Angas | Ah16 | 9 | 9 | Plant-based agriculture |
| Garo | Ei1 | 26 | 91 | Plant-based agriculture |
| Toda | Eg4 | 12 | 77 | Animal husbandry |
| Aua | Ig13 | -1.74 | 142.85 | Foraging |
| Bontok | Ia8 | 17 | 121 | Plant-based agriculture |
| Choiseulese | Ig12 | -6.69 | 156.52 | Plant-based agriculture |
| Kubu | Ib8 | -3 | 103 | Foraging |
| Mangarevans | Ij7 | -23.12 | -134.97 | Foraging |
| Merina | Eh2 | -19 | 46 | Plant-based agriculture |
| Nauruans | If13 | -0.53 | 166.92 | Plant-based agriculture |
| Raroians | Ij5 | -15.94 | -142.31 | Foraging |
| Ulawans | Ig6 | -9.63 | 161.44 | Plant-based agriculture |
| Mekeo | Ie22 | -9 | 147 | Plant-based agriculture |
| Miriam | Ie14 | -9.91 | 144.06 | Plant-based agriculture |
| Aranda | Id1 | -24 | 134 | Foraging |
| Dieri | Id4 | -28 | 138 | Foraging |
| Gheg | Ce1 | 42 | 20 | Plant-based agriculture |
| Vedda | Eh4 | 8 | 81 | Foraging |
| Armenians | Ci10 | 40 | 45 | Plant-based agriculture |
| Selkup | Ec11 | 59 | 90 | Foraging |
| Yurak | Ec4 | 68 | 75 | Foraging |
| Koryak | Ec5 | 61.99 | 164.24 | Foraging |
| Caribou Inuit | Na21 | 63 | -96 | Foraging |
| Mistassini | Na45 | 52 | -72 | Foraging |
| Cree Attawapiskat | Na7 | 53 | -83 | Foraging |
| Tewa | Nh11 | 36 | -106 | Plant-based agriculture |
| Paez | Sf5 | 3 | -76 | Plant-based agriculture |
| Wapishana | Sc5 | 3 | -60 | Foraging |
| Ainu | Ec7 | 44 | 144 | Foraging |
| Alacaluf | Sg5 | -52 | -74 | Foraging |
| Guato | Si6 | -19 | -58 | Foraging |
| Ket | Ec8 | 62 | 90 | Foraging |
| Seri | Ni4 | 29 | -112 | Foraging |
| Moldovans | ch25 | 47 | 29 | Plant-based agriculture |
| Veps | ch27 | 61 | 35 | Plant-based agriculture |
| Votes | ch28 | 59 | 29 | Plant-based agriculture |
| Lithuanian Tatar | ch23 | 55 | 24 | Plant-based agriculture |
| Chuvash | ch14 | 57 | 53 | Plant-based agriculture |
| Estonians | ch16 | 59 | 26 | Plant-based agriculture |
| Mambwe | Ac43 | 9 | 32 | Plant-based agriculture |
| Hatsa Kindiga | Aa9 | -3 | 35 | Foraging |
| Naron | Aa7 | -20 | 24 | Foraging |
| Bassari | Ag21 | 12 | -13 | Plant-based agriculture |
| Coniagui | Ag8 | 13 | -13 | Plant-based agriculture |
| Bunda | Ac21 | -5 | 19 | Plant-based agriculture |
| Buye | Ac31 | -7 | 28 | Plant-based agriculture |
| Temne | Af57 | 8.46 | -12.64 | Plant-based agriculture |
| Babwa | Ae7 | 3 | 25 | Plant-based agriculture |
| Banyang | Af16 | 6 | 9 | Plant-based agriculture |
| Bende | Ad18 | -7 | 31 | Plant-based agriculture |
| Chagga | Ad3 | -3 | 37 | Plant-based agriculture |
| Dzem | Ae40 | 3 | 14 | Plant-based agriculture |
| Fipa | Ad19 | -8 | 31 | Plant-based agriculture |
| Hehe | Ad8 | -8 | 35 | Plant-based agriculture |
| Ibibio | Af20 | 5 | 8 | Plant-based agriculture |
| Kamba | Ad34 | -2 | 38 | Plant-based agriculture |
| Bamileke | Ae5 | 5 | 10 | Plant-based agriculture |
| Kuba | Ac4 | -5 | 22 | Plant-based agriculture |
| Nyanja | Ac38 | -16 | 36 | Plant-based agriculture |
| Ruanda | Ae10 | -2 | 30 | Plant-based agriculture |
| Rundi | Ae8 | -3 | 30 | Plant-based agriculture |
| Sakata | Ac24 | -3 | 18 | Plant-based agriculture |
| Shambala | Ad10 | -5 | 38 | Plant-based agriculture |
| Suku | Ac17 | -6 | 18 | Plant-based agriculture |
| Sundi | Ac18 | -5 | 14 | Plant-based agriculture |
| Tikar | Ae58 | 6 | 12 | Plant-based agriculture |
| Topoke | Ae26 | 0 | 24 | Plant-based agriculture |
| Wute | Ah8 | 6 | 12 | Plant-based agriculture |
| Baya | Ai7 | 6 | 16 | Plant-based agriculture |
| Mumuye | Ah31 | 9 | 12 | Plant-based agriculture |
| Awuna | Ag37 | 11 | -2 | Plant-based agriculture |
| Bobo | Ag30 | 13 | -4 | Plant-based agriculture |
| Builsa | Ag38 | 11 | -2 | Plant-based agriculture |
| Dagari | Ag39 | 11 | -3 | Plant-based agriculture |
| Dagomba | Ag44 | 9 | 0 | Plant-based agriculture |
| Kasena | Ag13 | 11 | -1 | Plant-based agriculture |
| Kulango | Ag35 | 7 | -3 | Plant-based agriculture |
| Lobi | Ag11 | 10 | -4 | Plant-based agriculture |
| Minianka | Ag31 | 12 | -6 | Plant-based agriculture |
| Moba | Ag50 | 11 | 1 | Plant-based agriculture |
| Nankanse | Ag12 | 10 | -1 | Plant-based agriculture |
| Nunuma | Ag42 | 12 | -3 | Plant-based agriculture |
| Gbari | Af28 | 10 | 10 | Plant-based agriculture |
| Diula | Ag27 | 9 | -4 | Plant-based agriculture |
| Koranko | Ag24 | 9 | -11 | Plant-based agriculture |
| Samo | Ag29 | 13 | -3 | Plant-based agriculture |
| Yalunka | Ag54 | 10 | -12 | Plant-based agriculture |
| Popoi | Ai28 | 2 | 26 | Plant-based agriculture |
| Hill Suk | Aj26 | 1 | 36 | Plant-based agriculture |
| Antessar | Cc5 | 18 | -3 | Animal husbandry |
| Jebala | Cd15 | 35 | -5 | Plant-based agriculture |
| Kababish | Cc6 | 17 | 31 | Animal husbandry |
| Kunta | Cc18 | 19 | -1 | Animal husbandry |
| Zenaga | Cc20 | 18 | -8 | Animal husbandry |
| Angami | Ei13 | 26 | 94 | Plant-based agriculture |
| Karen | Ei7 | 17.12 | 97.19 | Plant-based agriculture |
| Kalinga | Ia16 | 18 | 121 | Plant-based agriculture |
| Ponapeans | If5 | 6.88 | 158.22 | Plant-based agriculture |
| Toradja | Ic5 | -2 | 121 | Plant-based agriculture |
| Purari | Ie8 | -7 | 145 | Foraging |
| Tobelorese | Ic10 | 1 | 128 | Plant-based agriculture |
| Kariera | Id5 | -21 | 118 | Foraging |
| Wongaibon | Id9 | -32 | 146 | Foraging |
| Byelorussians | Ch6 | 55 | 28 | Plant-based agriculture |
| Kazak | Eb1 | 48 | 70 | Animal husbandry |
| Turkmen | Eb5 | 38 | 62 | Plant-based agriculture |
| Greenlanders | Na25 | 68.66 | -51.22 | Foraging |
| Taqagmiut | Na44 | 62 | -76 | Foraging |
| Taulipang | Sc15 | 4 | -62 | Foraging |
| Witoto | Se6 | -1 | -74 | Foraging |
| Aweikoma | Sj3 | -28 | -50 | Foraging |
| Choroti | Sh5 | -22 | -62 | Foraging |
| Zuni | Nh4 | 35 | -109 | Plant-based agriculture |
| Kazan tatar | ch20 | 55 | 50 | Plant-based agriculture |
| Ingassana | Ai4 | 11 | 34 | Plant-based agriculture |
| Anaguta | Ah9 | 10 | 9 | Plant-based agriculture |
| Bamum | Ae50 | 6 | 11 | Plant-based agriculture |
| Basakomo | Ah12 | 8 | 7 | Plant-based agriculture |
| Bira | Ae30 | 1 | 29 | Plant-based agriculture |
| Bombesa | Ae36 | 1 | 23 | Plant-based agriculture |
| Bubi | Ae44 | 3.54 | 8.72 | Plant-based agriculture |
| Budu | Ae31 | 2 | 28 | Plant-based agriculture |
| Chawai | Ah10 | 10 | 9 | Plant-based agriculture |
| Ndoko | Ae38 | 2 | 21 | Plant-based agriculture |
| Gure | Ah6 | 10 | 8 | Plant-based agriculture |
| Ila | Ac1 | -16 | 27 | Plant-based agriculture |
| Jukun | Ah2 | 8 | 10 | Plant-based agriculture |
| Kagoro | Ah20 | 10 | 8 | Plant-based agriculture |
| Kurama | Ah21 | 10 | 9 | Plant-based agriculture |
| Lamba | Ac5 | -13 | 28 | Plant-based agriculture |
| Mambila | Ah4 | 7 | 12 | Plant-based agriculture |
| Ndaka Babali | Ae33 | 1 | 27 | Plant-based agriculture |
| Ndoro | Ah26 | 7 | 11 | Plant-based agriculture |
| Ngombe | Ae39 | 2 | 20 | Plant-based agriculture |
| Ngonde | Ad16 | -10 | 34 | Plant-based agriculture |
| Nyamwezi | Ad20 | -5 | 33 | Plant-based agriculture |
| Pende | Ac2 | -6 | 20 | Plant-based agriculture |
| Plains Bira | Ae34 | 1 | 30 | Plant-based agriculture |
| Safwa | Ad17 | -8 | 33 | Plant-based agriculture |
| Tigon | Ah25 | 17 | 11 | Plant-based agriculture |
| Tumbuka | Ac36 | -12 | 34 | Plant-based agriculture |
| Yaka | Ac20 | -7 | 17 | Plant-based agriculture |
| Yao | Ac7 | -13 | 36 | Plant-based agriculture |
| Dan | Af50 | 8 | 8 | Plant-based agriculture |
| Zuande | Ah27 | 7 | 11 | Plant-based agriculture |
| Azande | Ai3 | 5 | 27 | Plant-based agriculture |
| Banda | Ai1 | 7 | 22 | Plant-based agriculture |
| Bwaka | Ai23 | 3 | 19 | Plant-based agriculture |
| Chamba | Ah28 | 8 | 11 | Plant-based agriculture |
| Longuda | Ah30 | 10 | 12 | Plant-based agriculture |
| Ngbandi | Ai26 | 4 | 22 | Plant-based agriculture |
| Vere | Ah32 | 9 | 13 | Plant-based agriculture |
| Yungur | Ah33 | 10 | 12 | Plant-based agriculture |
| Kusasi | Ag41 | 11 | 0 | Plant-based agriculture |
| Manprusi | Ag46 | 10 | -1 | Plant-based agriculture |
| Egba | Af32 | 7 | 3 | Plant-based agriculture |
| Igala | Af30 | 7 | 7 | Plant-based agriculture |
| Daka | Ah29 | 8 | 11 | Plant-based agriculture |
| Kpelle | Af15 | 7 | -9 | Plant-based agriculture |
| Toma | Af11 | 11 | -9 | Plant-based agriculture |
| Lendu | Ai29 | 2 | 30 | Plant-based agriculture |
| Lese | Ai30 | 2 | 29 | Plant-based agriculture |
| Logo | Ai31 | 3 | 30 | Plant-based agriculture |
| Mamvu | Ai5 | 3 | 29 | Plant-based agriculture |
| Anuak | Ai44 | 8 | 34 | Plant-based agriculture |
| Dorobo | Aa2 | 0 | 36 | Foraging |
| Jur | Ai36 | 8 | 28 | Plant-based agriculture |
| Liptako | Cb22 | 14 | 0 | Plant-based agriculture |
| Bororo Fulani | Cb8 | 13 | 5 | Animal husbandry |
| Barabra | Cd1 | 23 | 33 | Plant-based agriculture |
| Mzab | Cc4 | 33 | 4 | Plant-based agriculture |
| Shluh | Cd5 | 30 | -9 | Plant-based agriculture |
| Siwans | Cc3 | 29 | 26 | Plant-based agriculture |
| Udalan | Cc13 | 16 | 0 | Plant-based agriculture |
| Ababda | Ca34 | 24 | 34 | Animal husbandry |
| Janjero | Ca29 | 8 | 38 | Plant-based agriculture |
| Bachama | Cb27 | 10 | 12 | Plant-based agriculture |
| Bata | Ah34 | 10 | 13 | Plant-based agriculture |
| Bolewa | Cb7 | 11 | 11 | Plant-based agriculture |
| Bura | Ah35 | 10 | 12 | Plant-based agriculture |
| Dera | Cb28 | 10 | 12 | Plant-based agriculture |
| Gude | Ah36 | 10 | 13 | Plant-based agriculture |
| Hona | Ah37 | 10 | 13 | Plant-based agriculture |
| Karekare | Cb10 | 12 | 11 | Plant-based agriculture |
| Tera | Cb6 | 11 | 12 | Plant-based agriculture |
| Nail | Cd17 | 35 | 5 | Animal husbandry |
| Rwala | Cj2 | 33 | 37 | Animal husbandry |
| Saadi | Cd18 | 30 | 28 | Animal husbandry |
| Sahel | Cd19 | 35 | 10.94 | Plant-based agriculture |
| Shuwa | Cb16 | 13 | 15 | Animal husbandry |
| Ao | Ei14 | 27 | 94 | Plant-based agriculture |
| Tibetans | Ee4 | 30 | 91 | Plant-based agriculture |
| Palaung | Ei18 | 23 | 97 | Plant-based agriculture |
| Semang | Ej3 | 5 | 101 | Foraging |
| Dobuans | Ig5 | -10 | 151 | Plant-based agriculture |
| Ifugao | Ia3 | 17 | 121 | Plant-based agriculture |
| Kaoka | Ig20 | -9.49 | 159.82 | Plant-based agriculture |
| Manobo | Ia15 | 8 | 126 | Plant-based agriculture |
| Manus | Ig9 | -2 | 147 | Foraging |
| Mentaweians | Ib7 | -2.93 | 100.23 | Plant-based agriculture |
| Minangkabau | Ib6 | -1 | 101 | Plant-based agriculture |
| Samoans | Ii1 | -13.68 | -172.41 | Plant-based agriculture |
| Selung | Ej6 | 11.78 | 98.28 | Foraging |
| Tannese | Ih10 | -19.6 | 169.44 | Plant-based agriculture |
| Tongans | Ii12 | -19.74 | -175.07 | Plant-based agriculture |
| Elema | Ie39 | -7.82 | 145.31 | Foraging |
| Min Chinese | Ed6 | 24 | 115 | Plant-based agriculture |
| Rossel | Ig11 | -11.36 | 154.14 | Foraging |
| Wantoat | Ie2 | -6.15 | 146.53 | Plant-based agriculture |
| Tiwi | Id3 | -11.56 | 130.81 | Foraging |
| Wikmunkan | Id6 | -14 | 142 | Foraging |
| New England | Cf1 | 42 | -73 | Plant-based agriculture |
| Bakhtiari | Ea8 | 33 | 48 | Animal husbandry |
| Mafulu | Ie25 | -8 | 147 | Plant-based agriculture |
| Goldi | Ec9 | 47 | 132 | Foraging |
| Kalmyk | Ci1 | 46 | 46 | Animal husbandry |
| Monguor | Eb2 | 39 | 100 | Plant-based agriculture |
| Sivokakmeit | Na11 | 63.34 | -170.3 | Foraging |
| Copper Inuit | Na3 | 69 | -110 | Foraging |
| Netsilik | Na43 | 69 | -96 | Foraging |
| Iglulik | Na22 | 70 | -82 | Foraging |
| Kaska | Na4 | 59 | -128 | Foraging |
| Acoma | Nh13 | 35 | -108 | Plant-based agriculture |
| Laguna | Nh14 | 35 | -107 | Plant-based agriculture |
| Santa Ana | Nh12 | 35 | -107 | Plant-based agriculture |
| Sia | Nh25 | 36 | -107 | Plant-based agriculture |
| Hopi | Nh18 | 36 | -111 | Plant-based agriculture |
| Isleta | Nh10 | 35 | -107 | Plant-based agriculture |
| Picuris | Nh9 | 36 | -106 | Plant-based agriculture |
| Jemez | Nh8 | 36 | -107 | Plant-based agriculture |
| Yekuana | Sc16 | 3 | -65 | Foraging |
| Miskito | Sa9 | 13 | -85 | Foraging |
| Bororo | Si1 | -16 | -55 | Foraging |
| Conibo | Se9 | -9 | -74 | Foraging |
| Gilyak | Ec1 | 53 | 142 | Foraging |
| Ingrians | ch18 | 59 | 29 | Plant-based agriculture |
| Korongo | Ai38 | 10 | 30 | Plant-based agriculture |
| Tira | Ai41 | 11 | 30 | Plant-based agriculture |
| Balante | Ag15 | 12 | -16 | Plant-based agriculture |
| Banyune | Ag16 | 12 | -16 | Plant-based agriculture |
| Sherbro | Af14 | 7.52 | -12.69 | Plant-based agriculture |
| Bafia | Ae48 | 5 | 11 | Plant-based agriculture |
| Vugusu | Ad41 | 1 | 35 | Plant-based agriculture |
| Bena | Ad11 | -9 | 36 | Plant-based agriculture |
| Birom | Ah17 | 10 | 9 | Plant-based agriculture |
| Chiga | Ad13 | -3 | 30 | Plant-based agriculture |
| Dakakari | Ah13 | 12 | 5 | Plant-based agriculture |
| Groote Eylandt | Id13 | -13.99 | 136.6 | Foraging |
| Hadimu | Ad29 | -6.07 | 39.43 | Plant-based agriculture |
| Kadara | Ah19 | 10 | 8 | Plant-based agriculture |
| Kikuyu | Ad4 | -1 | 37 | Plant-based agriculture |
| Kisama | Ab21a | -10 | 14 | Plant-based agriculture |
| Lovedu | Ab14 | -24 | 31 | Plant-based agriculture |
| Luchazi | Ac27 | -13 | 23 | Plant-based agriculture |
| Luguru | Ad14 | -8 | 38 | Plant-based agriculture |
| Luimbe | Ac28 | -12 | 18 | Foraging |
| Luvale | Ac11 | -12 | 22 | Plant-based agriculture |
| Mbuti | Aa5 | 2 | 28 | Foraging |
| Ndembu | Ac6 | -11 | 26 | Plant-based agriculture |
| Ngulu | Ad51 | -6 | 38 | Plant-based agriculture |
| Nkundo | Ae4 | 0 | 20 | Plant-based agriculture |
| Nsungli | Ae57 | 6 | 11 | Plant-based agriculture |
| Nyakyusa | Ad6 | -9 | 34 | Plant-based agriculture |
| Pimbwe | Ad21 | -7 | 31 | Plant-based agriculture |
| Yako | Af4 | 6 | 8 | Plant-based agriculture |
| Ngere | Af56 | 7 | -8 | Plant-based agriculture |
| Birifor | Ag5 | 10 | -3 | Plant-based agriculture |
| Dogon | Ag3 | 15 | -3 | Plant-based agriculture |
| Dorosie | Ag34 | 11 | -4 | Plant-based agriculture |
| Gurma | Ag45 | 12 | 2 | Plant-based agriculture |
| Kabre | Ag49 | 10 | 2 | Plant-based agriculture |
| Akyem | Af12 | 7 | -1 | Plant-based agriculture |
| Tallensi | Ag4 | 11 | -1 | Plant-based agriculture |
| Ibo | Af10 | 6 | 7 | Plant-based agriculture |
| Ife | Af34 | 7 | 5 | Plant-based agriculture |
| Nupe | Af8 | 9 | 6 | Plant-based agriculture |
| Bozo | Ag7 | 14 | -5 | Foraging |
| Gbande | Af54 | 8 | -10 | Plant-based agriculture |
| Malinke | Ag9 | 11 | -9 | Plant-based agriculture |
| Mende | Af5 | 8 | -11 | Plant-based agriculture |
| Meban | Ai45 | 10 | 34 | Plant-based agriculture |
| Susu | Ag26 | 15 | -13 | Plant-based agriculture |
| Fajulu | Aj13 | 4 | 31 | Plant-based agriculture |
| Jie | Aj21 | 3 | 34 | Plant-based agriculture |
| Labwor | Aj22 | 3 | 34 | Plant-based agriculture |
| Nyima | Ai43 | 12 | 29 | Plant-based agriculture |
| Kanembu | Cb18 | 14 | 14 | Plant-based agriculture |
| Koma | Ai46 | 9 | 35 | Plant-based agriculture |
| Amarar | Ca35 | 20 | 36 | Animal husbandry |
| Anfillo | Ca27 | 9 | 35 | Plant-based agriculture |
| Bisharin | Ca5 | 20 | 35 | Animal husbandry |
| Darasa | Ca15 | 6 | 38 | Plant-based agriculture |
| Jimma | Ca39 | 8 | 37 | Plant-based agriculture |
| Konso | Ca1 | 6 | 37 | Plant-based agriculture |
| Sidamo | Ca16 | 7 | 38 | Plant-based agriculture |
| Gisiga | Ai17 | 10 | 14 | Plant-based agriculture |
| Kapsiki | Ah38 | 11 | 14 | Plant-based agriculture |
| Margi | Ah5 | 11 | 13 | Plant-based agriculture |
| Tazarawa | Cb25 | 14 | 8 | Plant-based agriculture |
| Chaambra | Cc16 | 32 | 3 | Animal husbandry |
| Druze | Cj8 | 32.77 | 35.03 | Plant-based agriculture |
| Hamama | Cd13 | 30 | 9 | Animal husbandry |
| Moroccans | Cd16 | 33 | -8 | Plant-based agriculture |
| Mutair | Cj5 | 28 | 47 | Animal husbandry |
| Tunisians | Cd21 | 37 | 10 | Plant-based agriculture |
| Lakher | Ei4 | 22 | 93 | Plant-based agriculture |
| Lepcha | Ee3 | 28 | 89 | Plant-based agriculture |
| Minchia | Ed8 | 26 | 100 | Plant-based agriculture |
| Purum | Ei6 | 25 | 94 | Plant-based agriculture |
| Coorg | Eg5 | 12 | 76 | Plant-based agriculture |
| Baiga | Eg9 | 22 | 81 | Plant-based agriculture |
| Chahar | Eb7 | 41 | 115 | Animal husbandry |
| Shantung | Ed10 | 37 | 118 | Plant-based agriculture |
| Ami | Ia9 | 22.7 | 121.05 | Plant-based agriculture |
| Atayal | Ia1 | 24 | 121 | Plant-based agriculture |
| Batak | Ib4 | 2 | 99 | Plant-based agriculture |
| Bunun | Ia10 | 24 | 121 | Plant-based agriculture |
| Manam | Ie29 | -4.08 | 145.03 | Plant-based agriculture |
| Paiwan | Ia6 | 22.44 | 120.77 | Plant-based agriculture |
| Puyuma | Ia11 | 23 | 121 | Plant-based agriculture |
| Sumbanese | Ic9 | -10 | 120 | Plant-based agriculture |
| Tanala | Eh3 | -22 | 47 | Plant-based agriculture |
| Tangu | Ig21 | -3.38 | 153.31 | Plant-based agriculture |
| Waropen | Ie6 | -2.14 | 137.23 | Foraging |
| Abelam | Ie15 | -3.79 | 143.09 | Plant-based agriculture |
| Arapesh | Ie3 | -4 | 144 | Plant-based agriculture |
| Iatmul | Ie35 | -4 | 143 | Plant-based agriculture |
| Li | Ed9 | 19 | 109 | Plant-based agriculture |
| Murngin | Id2 | -12.48 | 135.96 | Foraging |
| Walbiri | Id10 | -22 | 132 | Foraging |
| Yir Yoront | Id12 | -15 | 142 | Foraging |
| French Canadians | Cf5 | 47 | -72 | Plant-based agriculture |
| Haitians | Sb9 | 18.41 | -72.17 | Plant-based agriculture |
| Ukranians | Ch7 | 48 | 36 | Plant-based agriculture |
| Keraki | Ie5 | -9 | 142 | Plant-based agriculture |
| Chugach | Na10 | 60.72 | -146.49 | Foraging |
| Nunivak | Na6 | 60 | -166 | Foraging |
| Nabesna | Na1 | 63 | -141 | Foraging |
| Chorti | Sa3 | 14 | -89 | Plant-based agriculture |
| Quiche | Sa13 | 15 | -91 | Plant-based agriculture |
| Mixe | Nj7 | 17 | -95 | Plant-based agriculture |
| Camaracoto | Sc11 | 6 | -63 | Plant-based agriculture |
| Barama Carib | Sc3 | 5 | -59 | Foraging |
| Ramcocamecra | Sj4 | -7 | -45 | Foraging |
| Burusho | Ee2 | 37 | 75 | Plant-based agriculture |
| Jivaro | Se3 | -3 | -78 | Plant-based agriculture |
| Trumai | Si2 | -12 | -53 | Foraging |
| Nganasan | ec12 | 72 | 90 | Foraging |
| Kakwa | Aj14 | 4 | 31 | Plant-based agriculture |
| Chekiang | Ed15b | 31 | 120 | Plant-based agriculture |
| Mao | Ai47 | 9 | 35 | Plant-based agriculture |
| Murinbata | Id7 | -14 | 130 | Foraging |
| Motilon | Sb3 | 9 | -72 | Foraging |
| Koalib | Ai37 | 12 | 31 | Plant-based agriculture |
| Mesakin | Ai39 | 11 | 30 | Plant-based agriculture |
| Moro | Ai40 | 11 | 30 | Plant-based agriculture |
| Otoro | Ai10 | 12 | 31 | Plant-based agriculture |
| Tullishi | Ai42 | 12 | 29 | Plant-based agriculture |
| Biafada | Ag17 | 12 | -15 | Plant-based agriculture |
| Banen | Ae51 | 5 | 11 | Plant-based agriculture |
| Duala | Ae12 | 4 | 10 | Plant-based agriculture |
| Gusii | Ad12 | -1 | 35 | Plant-based agriculture |
| Kota | Ae41 | 1 | 14 | Plant-based agriculture |
| Lala | Ac33 | -15 | 31 | Plant-based agriculture |
| Meru | Ad35 | 0 | 35 | Plant-based agriculture |
| Ngoni | Ac9 | -12 | 33 | Plant-based agriculture |
| Plateau Tonga | Ac30 | -18 | 28 | Plant-based agriculture |
| Shogo | Ac16 | -1 | 12 | Plant-based agriculture |
| Teita | Ad37 | -4 | 39 | Plant-based agriculture |
| Konkomba | Ag10 | 10 | 0 | Plant-based agriculture |
| Somba | Ag51 | 11 | 1 | Plant-based agriculture |
| Adangme | Af35 | 6 | 0 | Plant-based agriculture |
| Itsekiri | Af22 | 6 | 5 | Foraging |
| Kran | Af47 | 6 | -8 | Plant-based agriculture |
| Sapo | Af49 | 6 | -9 | Plant-based agriculture |
| Luo | Aj6 | -1 | 34 | Plant-based agriculture |
| Tatoga | Aj28 | -5 | 35 | Animal husbandry |
| Arbore | Ca18 | 5 | 37 | Plant-based agriculture |
| Hausa Kanawa | Cb9 | 12 | 9 | Plant-based agriculture |
| Matakam | Ah7 | 11 | 14 | Plant-based agriculture |
| Gurage | Ca8 | 8 | 38 | Plant-based agriculture |
| Messiria Humr | Cb15 | 11 | 28 | Animal husbandry |
| Sanusi | Cd20 | 31 | 22 | Animal husbandry |
| Chin | Ei19 | 22 | 94 | Plant-based agriculture |
| Kachin | Ei5 | 26 | 97 | Plant-based agriculture |
| Quahgai | Ea12 | 30 | 52 | Animal husbandry |
| Chenchu | Eg1 | 16 | 79 | Foraging |
| Khalka | Eb3 | 46 | 97 | Animal husbandry |
| Oraon | Ef6 | 23 | 85 | Plant-based agriculture |
| Reddi | Eg14 | 18 | 82 | Plant-based agriculture |
| Santal | Ef1 | 24 | 87 | Plant-based agriculture |
| Alorese | Ic2 | -8.22 | 124.26 | Plant-based agriculture |
| Busama | Ie28 | -6.69 | 147.26 | Plant-based agriculture |
| Gilbert Onotoa | If7 | -1.47 | 175.06 | Foraging |
| Macassarese | Ic1 | -5.31 | 119.69 | Plant-based agriculture |
| Malays | Ej8 | 5 | 103 | Plant-based agriculture |
| Vanua Levu | Ih8 | -17.81 | 178.32 | Plant-based agriculture |
| Lolo | Ed2 | 29 | 103 | Plant-based agriculture |
| Siane | Ie17 | -6 | 145 | Plant-based agriculture |
| Kol | Eg8 | 22 | 85 | Plant-based agriculture |
| Siamese | Ej9 | 15 | 100 | Plant-based agriculture |
| Kimam | Ie18 | -7.52 | 138.46 | Plant-based agriculture |
| Bengali | Ef2 | 23 | 88 | Plant-based agriculture |
| Brazilians | Cf4 | -23.68 | -46.64 | Plant-based agriculture |
| Bulgarians | Ch5 | 43 | 24 | Plant-based agriculture |
| Czechs | Ch3 | 50 | 16 | Plant-based agriculture |
| Hungarian | Ch8 | 47 | 20 | Plant-based agriculture |
| Ngarawapum | Ie26 | -6 | 146 | Plant-based agriculture |
| Miao | Ed4 | 26 | 107 | Plant-based agriculture |
| Pekangekum | Na34 | 52 | -94 | Foraging |
| Popoluca | Nj3 | 18 | -95 | Plant-based agriculture |
| Black Carib | Sa7 | 16 | -89 | Plant-based agriculture |
| Cagaba | Sb2 | 11 | -74 | Plant-based agriculture |
| Yagua | Se4 | -3 | -72 | Foraging |
| Cuna | Sa1 | 9 | -78 | Plant-based agriculture |
| Caduveo | Sh4 | -22 | -57 | Foraging |
| Camayura | Si5 | -12 | -54 | Foraging |
| Siriono | Se1 | -16 | -64 | Foraging |
| Goajiro | Sb6 | 12 | -72 | Animal husbandry |
| Chinantec | Nj1 | 18 | -96 | Plant-based agriculture |
| Nambicura | Si4 | -12 | -59 | Foraging |
| Totonac | Nj4 | 20 | -97 | Plant-based agriculture |
| Tucuna | Se2 | -3 | -70 | Foraging |
| Uttar Pradesh | Ef11 | 26 | 83 | Plant-based agriculture |
| Lenca | Sa12 | 14 | -88 | Plant-based agriculture |
| Kung | Aa1 | -20 | 21 | Foraging |
| Gola | Af55 | 7 | -11 | Plant-based agriculture |
| Kissi | Af2 | 9 | -10 | Plant-based agriculture |
| Wodaabe Fulani | Cb24 | 12 | 12 | Animal husbandry |
| Wolof | Cb2 | 15 | -17 | Plant-based agriculture |
| Amba | Ae1 | 1 | 30 | Plant-based agriculture |
| Basa | Ah11 | 8 | 8 | Plant-based agriculture |
| Kunda | Ac37 | -15 | 32 | Plant-based agriculture |
| Lele | Ac23 | -5 | 21 | Plant-based agriculture |
| Ndob | Ae55 | 6 | 10 | Plant-based agriculture |
| Fut | Ae9 | 6 | 10 | Plant-based agriculture |
| Nsaw | Ae56 | 6 | 11 | Plant-based agriculture |
| Nyoro | Ad2 | 2 | 32 | Plant-based agriculture |
| Soga | Ad46 | 1 | 33 | Plant-based agriculture |
| Sonjo | Ad39 | -3 | 36 | Plant-based agriculture |
| Sukuma | Ad22 | -3 | 34 | Plant-based agriculture |
| Tiriki | Ad40 | 0 | 35 | Plant-based agriculture |
| Toro | Ad48 | 1 | 31 | Plant-based agriculture |
| Zinza | Ad49 | -3 | 31 | Plant-based agriculture |
| Fali | Ai12 | 10 | 14 | Plant-based agriculture |
| Tem | Ag52 | 11 | 1 | Plant-based agriculture |
| Yatenga | Ag2 | 13 | -2 | Plant-based agriculture |
| Afikpo | Af23 | 6 | 8 | Plant-based agriculture |
| Afo | Af27 | 8 | 8 | Plant-based agriculture |
| Bete | Af7 | 6 | -7 | Plant-based agriculture |
| Ekiti | Af33 | 8 | 5 | Plant-based agriculture |
| Idoma | Af29 | 7 | 8 | Plant-based agriculture |
| Oyo Yoruba | Af6 | 8 | 4 | Plant-based agriculture |
| Plains Suk | Aj23 | 1 | 36 | Animal husbandry |
| Samburu | Aj29 | 1 | 37 | Animal husbandry |
| Teso | Aj1 | 2 | 34 | Plant-based agriculture |
| Baditu | Ca40 | 6 | 38 | Plant-based agriculture |
| Bako | Ca23 | 6 | 37 | Plant-based agriculture |
| Basketo | Ca28 | 6 | 37 | Plant-based agriculture |
| Dime | Ca24 | 6 | 36 | Plant-based agriculture |
| Dorse | Ca41 | 6 | 38 | Plant-based agriculture |
| Macha | Ca13 | 9 | 37 | Plant-based agriculture |
| Shako | Ca42 | 7 | 36 | Plant-based agriculture |
| Shangama | Ca25 | 6 | 37 | Plant-based agriculture |
| Somali | Ca2 | 8 | 48 | Animal husbandry |
| Tsamai | Ca17 | 5 | 37 | Plant-based agriculture |
| Ubamer | Ca26 | 6 | 37 | Plant-based agriculture |
| Podokwo | Ah39 | 11 | 14 | Plant-based agriculture |
| Zazzagawa Hausa | Cb26 | 11 | 8 | Plant-based agriculture |
| Amhara | Ca7 | 13 | 38 | Plant-based agriculture |
| Barabish | Cc7 | 20 | -5 | Animal husbandry |
| Egyptians | Cd2 | 25 | 33 | Plant-based agriculture |
| Jordanians | Cj6 | 32 | 36 | Plant-based agriculture |
| Lebanese | Cj7 | 34 | 36 | Plant-based agriculture |
| Regeibat | Cc1 | 22 | -13 | Animal husbandry |
| Syrians | Cj1 | 36 | 36 | Plant-based agriculture |
| Tigrinya | Ca3 | 14 | 39 | Plant-based agriculture |
| Trarza | Cc19 | 18 | -15 | Animal husbandry |
| Yemeni | Cj9 | 15 | 45 | Plant-based agriculture |
| Burmese | Ei3 | 20 | 95 | Plant-based agriculture |
| Magar | Ee8 | 28 | 84 | Plant-based agriculture |
| Turks | Ci5 | 38 | 30 | Plant-based agriculture |
| Telugu | Eg10 | 18 | 79 | Plant-based agriculture |
| Cambodians | Ej5 | 12 | 105 | Plant-based agriculture |
| Annamese | Ej4 | 17 | 107 | Plant-based agriculture |
| Ambonese | Ic11 | -3.71 | 128.18 | Plant-based agriculture |
| Balinese | Ib3 | -8.18 | 115.02 | Plant-based agriculture |
| Belu | Ic3 | -9 | 126 | Plant-based agriculture |
| Bunlap | Ih3 | -15.95 | 168.22 | Plant-based agriculture |
| Cham | Ej11 | 11 | 108 | Plant-based agriculture |
| Chamorro | If8 | 15.13 | 145.71 | Plant-based agriculture |
| Hanunoo | Ia5 | 13 | 121 | Plant-based agriculture |
| Iban | Ib1 | 2 | 112 | Plant-based agriculture |
| Javanese | Ib2 | -7 | 110 | Plant-based agriculture |
| Rennell | Ii10 | -11.74 | 160.42 | Plant-based agriculture |
| Rhade | Ej10 | 13 | 108 | Plant-based agriculture |
| Sagada | Ia2 | 17 | 121 | Plant-based agriculture |
| Subanun | Ia4 | 8 | 123 | Plant-based agriculture |
| Sugbuhanon | Ia12 | 10.2 | 123.65 | Plant-based agriculture |
| Usiai | Ig10 | -2 | 147 | Plant-based agriculture |
| Enga | Ie7 | -6 | 144 | Plant-based agriculture |
| Fore | Ie31 | -6 | 145 | Plant-based agriculture |
| Kakoli | Ie11 | -6 | 144 | Plant-based agriculture |
| Kapauku | Ie1 | -4 | 136 | Plant-based agriculture |
| Mimika | Ie30 | -4 | 135 | Foraging |
| Gidjingali | Id11 | -12.29 | 134.32 | Foraging |
| Dutch | Cg1 | 53 | 7 | Plant-based agriculture |
| Greeks | Ce7 | 39 | 23 | Plant-based agriculture |
| Kashmir | Ef8 | 34 | 75 | Plant-based agriculture |
| Pahari | Ef7 | 30 | 78 | Plant-based agriculture |
| Punjabi | Ea13 | 32 | 73 | Plant-based agriculture |
| Sinhalese | Eh6 | 7 | 80 | Plant-based agriculture |
| Afghans | Ea11 | 33 | 68 | Plant-based agriculture |
| Iranians | Ea9 | 36 | 52 | Plant-based agriculture |
| Pathan | Ea2 | 35 | 72 | Plant-based agriculture |
| Spaniards | Ce6 | 37 | -6 | Plant-based agriculture |
| Walloons | Cg5 | 50 | 5 | Plant-based agriculture |
| Serbs | Ch1 | 44 | 20 | Plant-based agriculture |
| Lapps | Cg4 | 68 | 22 | Animal husbandry |
| Soromadja | Ie33 | -2 | 138 | Foraging |
| Japanese | Ed5 | 35 | 136 | Plant-based agriculture |
| Okinawans | Ed7 | 26.16 | 127.8 | Plant-based agriculture |
| Nunamiut | Na12 | 68 | -152 | Foraging |
| Hano | Nh2 | 36 | -111 | Plant-based agriculture |
| Carinya | Sb4 | 9 | -64 | Plant-based agriculture |
| Kuikuru | Si10 | -13 | -54 | Plant-based agriculture |
| Yabarana | Sc7 | 5 | -66 | Foraging |
| Tunebo | Sf4 | 7 | -72 | Plant-based agriculture |
| Waica | Sd4 | 2 | -65 | Foraging |
| Maue | Sd5 | -4 | -57 | Plant-based agriculture |
| Mundurucu | Sd1 | -6 | -58 | Foraging |
| Paraujano | Sb5 | 11 | -72 | Foraging |
| Piapoco | Sc17 | 6 | -68 | Plant-based agriculture |
| Coroa | Sj9 | -8 | -52 | Plant-based agriculture |
| Caraja | Sj1 | -12 | -50 | Foraging |
| Guahibo | Sc4 | 5 | -69 | Foraging |
| Koreans | Ed1 | 35 | 102 | Plant-based agriculture |
| Piaroa | Sc8 | 5 | -67 | Foraging |
| Sanema | Sd8 | 4 | -66 | Foraging |
| Warrau | Sc1 | 9 | -62 | Foraging |
| Sumbawanese | Ic12 | -8.63 | 117.27 | Plant-based agriculture |
| Russians | Ch11 | 53 | 41 | Plant-based agriculture |
| Negri Sembilan | Ej16 | 21 | 102 | Plant-based agriculture |
| Basseri | Ea6 | 30 | 34 | Animal husbandry |
| Shavante | Sj11 | -14 | -52 | Foraging |
| Bisa | Ag53 | 12 | 0 | Plant-based agriculture |
| Lawa | Ej12 | 18 | 98 | Plant-based agriculture |
| Senoi | Ej14 | 4 | 102 | Plant-based agriculture |
| Badjau Tawi-Tawi | Ia13 | 5.09 | 119.97 | Foraging |
| Dani | Ie38 | -4 | 139 | Plant-based agriculture |
| Djuka | Sc18 | 5 | -54 | Plant-based agriculture |
| Neapolitians | Ce5 | 40.89 | 14.25 | Plant-based agriculture |
| Chacobo | Se11 | -12 | -67 | Foraging |
| Choco | Sa4 | 8 | -78 | Plant-based agriculture |
| Shiriana | Sd6 | 4 | -63 | Foraging |
| Toba | Sh8 | -25 | -60 | Foraging |
| Yanomamo | Sd9 | 2 | -65 | Plant-based agriculture |


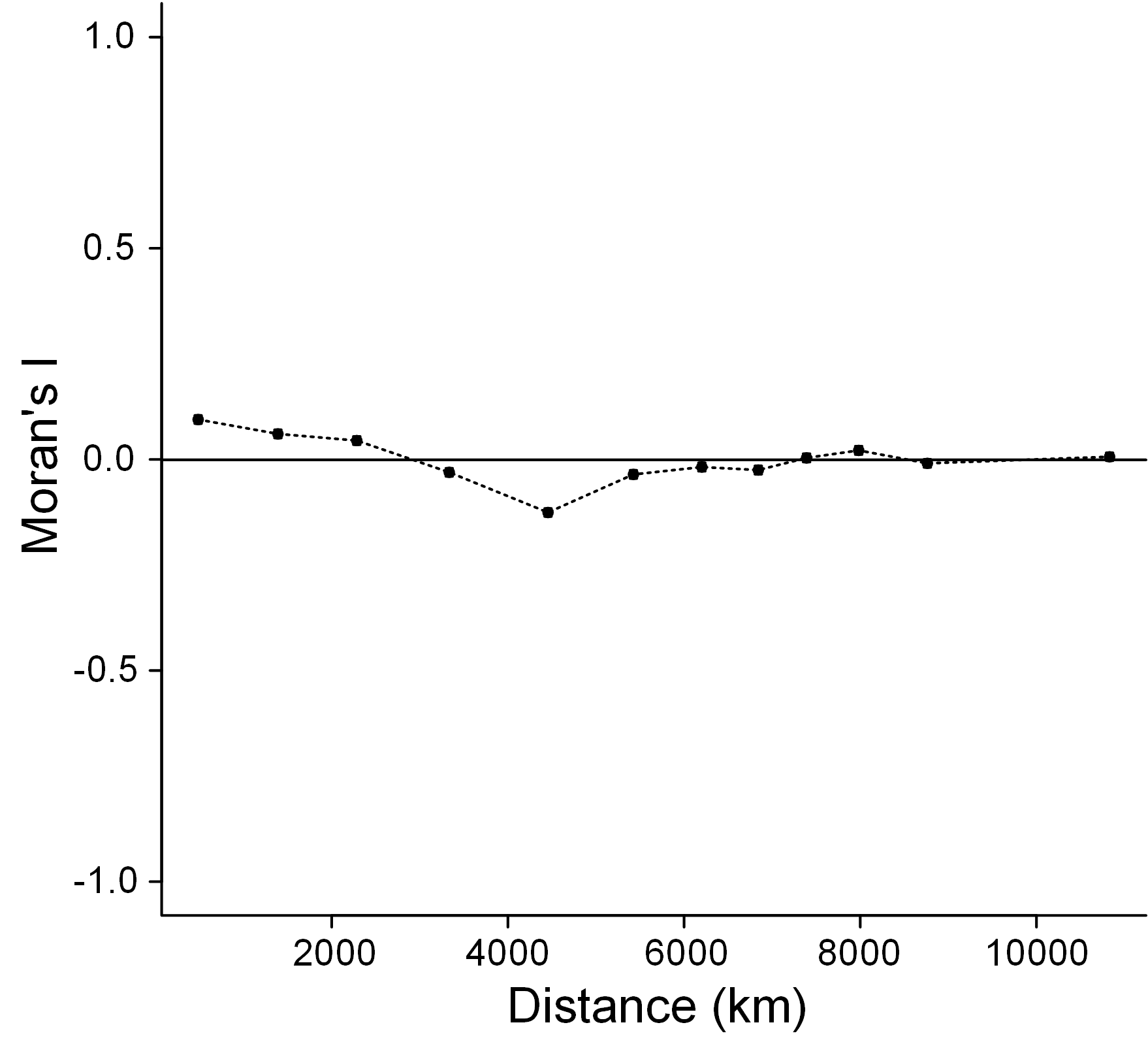


**Supplementary Figure 1. Moran’s I correlogram of model residuals by distance for the best-supported model of dominant subsistence strategy.** See Methods for details on sample and statistics.
